# Clinicopathological and immunohistochemical analysis of poorly differentiated squamous cell carcinoma and its impact on quality of life

**DOI:** 10.3389/fphar.2026.1755491

**Published:** 2026-05-25

**Authors:** Deepika Lakshmi Radhakrishnan, Gheena Sukumaran, Reena Das, Kavitha Ganesh, Najwa Abdur Rashid

**Affiliations:** 1 Department of Oral Pathology, SIMATS Deemed University Saveetha Dental College, Chennai, India; 2 Basic Science Department, College of Medicine, Princess Nourah bint Abdulrahman University, Riyadh, Saudi Arabia

**Keywords:** EGFR, mental health, oral cancer, disability, p63, poorly differentiated SCC, quality of life

## Abstract

**Background:**

Poorly differentiated squamous cell carcinoma (PDSCC) is an aggressive oral malignancy with rapid progression, high recurrence, and poor survival. This study aimed to evaluate the clinicopathological features, immunohistochemical expression of p63 and EGFR, and quality of life (QOL) in PDSCC compared to well-differentiated SCC (WDSCC).

**Methods:**

In this retrospective observational study, 78 histopathologically confirmed OSCC cases (39 PDSCC and 39 WDSCC) from 2019–2025 were analyzed. Histopathological parameters including tumor size, depth of invasion, perineural and lympho-vascular invasion, were recorded. Immunohistochemistry was performed on representative samples using p63 and EGFR. QOL was assessed using EORTC QLQ-C30 and QLQ-H&N43. Statistical analysis was performed using Chi-square/Fisher’s exact test and Mann-Whitney U test; survival was assessed using Kaplan-Meier and Log-rank test. Bonferroni correction was applied, with significance set at p < 0.001.

**Results:**

PDSCC showed shorter lesion duration compared to WDSCC. Significant differences were observed in anatomical site distribution, mitotic activity, and keratin pearl formation, reflecting the higher aggressiveness of PDSCC. Immunohistochemistry revealed reduced p63 and aberrant EGFR expression, though differences were not statistically significant. QOL was comparatively lower in PDSCC. Survival analysis confirmed inferior outcomes in PDSCC (mean survival 1.27 years) versus WDSCC (3.28 years, p < 0.001).

**Conclusion:**

PDSCC exhibits aggressive pathology and compromised QOL, underscoring the need for early recognition and biomarker-based prognostication.

## Introduction

Squamous cell carcinoma (SCC) is one of the most common malignancies of the head and neck region, with histological grading serving as a cornerstone for prognostic assessment and treatment planning (Mäkitie et al., 2022; Muthusamy et al., 2025; Srinivasan and Balasubramaniam, 2024; Yasothkumar et al., 2025). Based on GLOBOCAN 2020 data, cancers of the oral cavity and lip have an incidence rate of 10.2 per 100,000 population, with 377,713 new cases reported worldwide. Globally, oral squamous cell carcinoma (OSCC) is the eighth most common cancer, while in India it ranks third in prevalence (Poothakulath Krishnan et al., 2023). Among its variants, poorly differentiated squamous cell carcinoma (PDSCC) represents a particularly aggressive subtype, characterized by rapid growth, high recurrence rates, and reduced survival outcomes compared to well or moderately differentiated squamous cell carcinoma (WDSCC/MDSCC) (Padma et al., 2017). PDSCC exhibits adverse clinicopathological parameters, including increased lymph node metastasis, perineural and lymphovascular invasion, and deep tissue penetration (Rivera et al., 2014). Large tumor diameter (>2 cm) and invasion beyond subcutaneous fat are recognized as predictors of poor prognosis (Brancaccio et al., 2021). Histological subtypes such as desmoplastic and acantholytic SCC further carry metastasis risks as high as 21%–44%, underscoring the heterogeneity within this aggressive spectrum (Ciuciulete et al., 2021). Importantly, perineural invasion has been identified as an independent prognostic factor, with 5-year disease-specific survival dropping from 94.6% in its absence to 56.6% when present (Rahima et al., 2004). The diagnostic challenge lies in the lack of distinct squamous features in PDSCC, often necessitating the use of immunohistochemistry (IHC) for definitive diagnosis. Markers such as p63 and cytokeratin 5/6 have shown high sensitivity and specificity in distinguishing PDSCC from morphological mimics (Kaufmann et al., 2001). Additional markers, including CK34βE12, E-cadherin, Twist, and EGFR provide valuable insights into tumor proliferation, invasion, and epithelial-mesenchymal transition (Hasan et al., 2020; Scanlon et al., 2013; Zuo et al., 2011).

The Bryne scoring system has also been proposed as a superior alternative to conventional WHO grading, as it accounts for tumor heterogeneity and invasive front dynamics. Beyond histopathology and IHC, the impact of PDSCC on patients’ quality of life (QOL) is profound (Wang et al., 2013). Aggressive disease biology, radical surgical interventions, and adjuvant therapies often compromise global health status, physical functioning, nutrition, and social wellbeing. Psychosocial and financial burdens further exacerbate these outcomes. Factors such as low educational status, substance use, and lack of social support predict poorer QOL scores, emphasizing the need for a holistic evaluation of patient outcomes in addition to oncological control (Zahid et al., 2023). Despite these insights, few studies have comprehensively integrated clinicopathological evaluation, IHC profiling, and QOL assessment in PDSCC. Therefore, addressing this gap is essential for refining diagnostic strategies, prognostic frameworks, and patient-centered care. The present study aims to investigate the clinicopathological characteristics, immunohistochemical expression patterns of p63 and EGFR, and the quality of life outcomes in patients along with Kaplan-Meier survival analysis of poorly differentiated squamous cell carcinoma, in comparison with well-differentiated squamous cell carcinoma.

## Materials and methods

This retrospective observational study was conducted in the Department of Oral Pathology and Microbiology, Saveetha Dental College, SIMATS, Chennai, over a 6-year period from January 2019 to July 2025. This retrospective study was conducted in accordance with the principles of the Declaration of Helsinki. Ethical approval was obtained from the Institutional Scientific Review Board and Institutional Ethics Committee of Saveetha Dental College and Hospitals (Approval No: SRB/SDC/OPATH-2401/24/511). As the study primarily involved a retrospective analysis of archived histopathological records and paraffin-embedded tissue samples, the requirement for written informed consent was waived by the ethics committee. However, for quality of life assessment conducted through telephonic interviews, verbal informed consent was obtained from all participants prior to administration of the questionnaires. All procedures involving human participants were performed in accordance with institutional and ethical standards. Patient confidentiality and anonymity were strictly maintained throughout the study. The study population consisted of 39 histopathologically confirmed cases each of well-differentiated squamous cell carcinoma (WDSCC) and poorly differentiated squamous cell carcinoma (PDSCC). Out of these, five representative cases from each group were selected for detailed immunohistochemistry (IHC) analysis.

Cases were included if they had a confirmed histopathological diagnosis of well-differentiated squamous cell carcinoma (WDSCC) or poorly differentiated squamous cell carcinoma (PDSCC), sufficient paraffin-embedded tissue blocks for evaluation, and complete clinical records. Patients who had received chemotherapy or radiotherapy prior to biopsy, those with recurrent tumors or secondary carcinomas, and those with inadequately preserved tissue samples unsuitable for IHC were excluded. Clinical data such as age, sex, site of lesion, duration of symptoms, risk habits (tobacco, alcohol, betel nut chewing) and histopathological diagnosis were retrieved from case records. Histopathological parameters were assessed on hematoxylin and eosin-stained sections, including tumor diameter, depth of invasion (DOI), perineural invasion (PNI), lymphovascular invasion, bone infiltration, presence of dyskeratosis and mitotic index (expressed as MF/10 HPF). Tumors were graded according (Bryne et al., 1992).

For Immunohistochemistry (IHC) five paraffin-embedded blocks of poorly differentiated squamous cell carcinoma cases and five paraffin-embedded blocks of well differentiated squamous cell carcinoma cases from formalin-fixed, paraffin-embedded blocks were selected. Tissue sections of 3-µm thickness were sectioned and mounted on positively charged slides. To enhance tissue adhesion, the slides were incubated overnight at 37 °C and further incubated at 60 °C–70 °C for 20 min prior. Deparaffinization was achieved using two changes of xylene, followed by rehydration through graded alcohols and rinsing in distilled water. Antigen retrieval was performed in Tris-EDTA buffer (pH 8.5–9.0) using a pressure cooker for 10–15 min to unmask antigens from tissue, after which the sections were rinsed thoroughly in distilled water to remove excess buffer residues. Sections were then rinsed in PBS/TBS immunowash buffer for 2 min to equilibrate the tissue and prepare it for the subsequent peroxidase blocking step. Endogenous peroxidase activity was blocked by incubation with 3% hydrogen peroxide for 10 min. Wash in the wash buffer for changes 3 min each. The slides were then incubated with primary antibodies against p63 and EGFR for 45 min in a moist chamber to allow specific antigen–antibody binding, followed by buffer washes to remove unbound antibodies. To enhance detection, a polyExcel target binder reagent was applied for 12 min, after which an HRP-conjugated polymer secondary antibody was added and incubated for a further 12 min and wash with buffer for two changes 3 min each. Visualization of the antigen–antibody complex was accomplished using 3,3′-diaminobenzidine (DAB) chromogen, producing a brown reaction product at the antigen sites. The sections were then counterstained with hematoxylin for 30 s to provide nuclear contrast, dehydrated, cleared in xylene, and mounted with DPX. The stained slides were examined under light microscopy, and specific nuclear staining for p63 and membranous staining for EGFR were recorded for evaluation. Staining was scored semi-quantitatively using an immunoreactive score (IRS), based on both the proportion of positive cells and staining intensity. Aberrant expression patterns, such as cytoplasmic mislocalization of EGFR, were also documented. Two independent oral pathologists evaluated the slides, and any discrepancies were resolved by consensus to minimize inter-observer variation.

Quality of life assessment was integrated into the study design to provide a patient-centered perspective on clinical outcomes. Follow-up interviews were conducted using the European Organisation for Research and Treatment of Cancer (EORTC) QLQ-C30 core questionnaire, supplemented with the head and neck cancer specific module QLQ-H&N43 through telephonic calls. These tools provided a comprehensive evaluation of overall health status, physical and role functioning, psychosocial wellbeing and symptom burden, encompassing pain, dysphagia, xerostomia, weight loss, and challenges related to social functioning. Scores were transformed into a linear scale in accordance with EORTC guidelines, where higher functional scores indicated better quality of life and higher symptom scores denoted greater impairment.

For survival analysis, the year of death was recorded for all patients through follow-up calls. This information was used to calculate survival time and perform Kaplan-Meier analysis, with patients alive at last follow-up treated as censored observations.

All collected data were entered into Microsoft Excel and analyzed using IBM SPSS version 26 Statistics. Descriptive statistics were used to summarize demographic, clinicopathological, immunohistochemical, and quality of life variables. Categorical variables were compared between WDSCC and PDSCC groups using the Chi-square test or Fisher’s exact test, where appropriate. For immunohistochemical scoring, the data obtained for percentage of positive cells, staining intensity, and immunoreactive score (IRS) were assessed for normality. As the data were not normally distributed and the sample size was small, non-parametric statistical tests were employed. Descriptive statistics were expressed as median and interquartile range (IQR), and the Mann-Whitney U test was used to compare differences between the two independent groups for all quantitative variables. Quality of life scores derived from EORTC questionnaires were analyzed using the Mann–Whitney U test to compare scores between the two groups. Statistical significance was initially set at p ≤ 0.05, to control for Type I error due to multiple comparisons, Bonferroni correction was applied, yielding an adjusted significance threshold of p < 0.001. Survival outcomes were analyzed using the Kaplan-Meier survival analysis method, and differences between survival curves were assessed using the Log-rank test. A p-value of ≤0.05 was considered statistically significant.

## Results

### Clinicopathological parameters

A total of seventy-eight histopathologically confirmed cases of OSCC were analyzed, comprising well-differentiated squamous cell carcinoma (WDSCC, n = 39) and poorly differentiated squamous cell carcinoma (PDSCC, n = 39). Patients ranged in age from 20 to 92 years, with a mean age of 54.5 ± 12.5 years in the WDSCC group and 53.2 ± 10.8 years in the PDSCC group. Male predominance was observed in both groups, with 28 cases (71.8%) in WDSCC and 32 cases (82.1%) in PDSCC (p = 0.282). Medical comorbidities such as diabetes [7 cases (17.9%) vs. 6 cases (15.4%)], hypertension [2 cases (5.1%) vs. 4 cases (10.3%)], and other conditions showed no significant difference between WDSCC and PDSCC (p = 0.709). Habit history, including pan chewing [11 cases (28.2%) vs. 10 cases (25.6%)], smoking [4 cases (10.3%) vs. 3 cases (7.7%)], and combined habits [8 cases (20.5%) vs. 9 cases (23.1%)], was comparable between the groups (p = 0.940). Clinically, ulceroproliferative lesions were the most common presentation, observed in 23 cases (59.0%) of WDSCC and 20 cases (51.3%) of PDSCC, followed by ulcerative lesions [10 cases (25.6%) vs. 5 cases (12.8%)]. Pain and swelling were more frequent in PDSCC [10 cases (25.6%)] compared to WDSCC [3 cases (7.7%)], although not statistically significant (p = 0.137).

**FIGURE 1:**
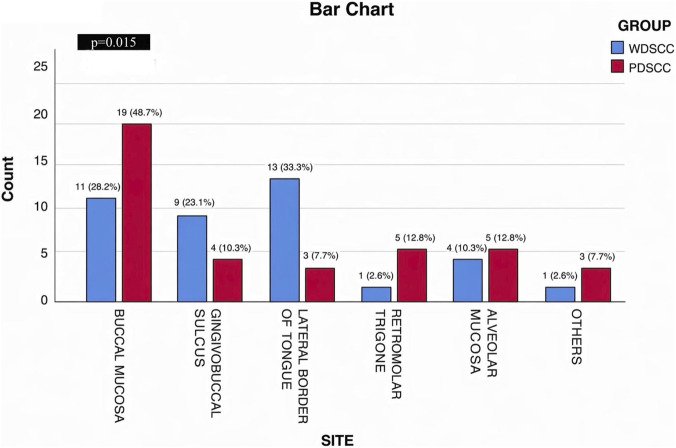
Comparison of site distribution between WDSCC and PDSCC groups. Statistical analysis was performed using the Chi-square test. Data are presented as frequency and percentage. P = 0.015 indicates a statistically significant difference.

Most cases presented within months, accounting for 23 cases (59.0%) in WDSCC and 22 cases (56.4%) in PDSCC, with no significant difference in duration (p = 0.097). Laterality showed a predominance of left-sided lesions [26 cases (66.7%) vs. 24 cases (61.5%)], with midline involvement seen only in PDSCC [4 cases (10.3%)] (p = 0.235). Site distribution demonstrated a statistically significant difference (p = 0.015), with buccal mucosa involvement higher in PDSCC [19 cases (48.7%)] compared to WDSCC [11 cases (28.2%)], while lateral border of tongue involvement was higher in WDSCC [13 cases (33.3%)] than PDSCC [3 cases (7.7%)] (Figure 1).

Bone invasion was absent in the majority of cases [34 cases (87.2%) vs. 35 cases (89.7%)], with no significant difference (p = 0.924). Skeletal muscle invasion was present in 5 cases (12.8%) of WDSCC and 8 cases (20.5%) of PDSCC (p = 0.362). Minor salivary gland involvement was absent in all WDSCC cases (0 cases, 0%) and present in 3 cases (7.7%) of PDSCC (p = 0.077). Lymphovascular invasion was present in 2 cases (5.1%) of WDSCC and 4 cases (10.3%) of PDSCC (p = 0.395), while perineural invasion was observed in 6 cases (15.4%) and 7 cases (17.9%), respectively (p = 0.761). Metastatic lymph node involvement was identified in 7 cases (17.9%) of WDSCC and 10 cases (25.6%) of PDSCC (p = 0.411). Extranodal extension (ENE), a marker of poor prognosis, was present in 2 cases (5.1%) in both groups (p = 0.820).

**FIGURE 2:**
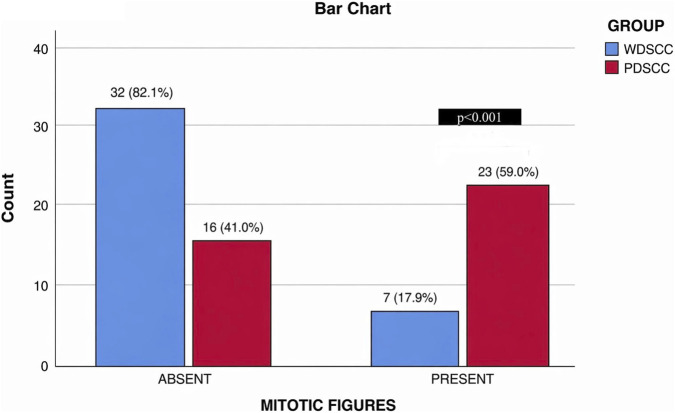
Comparison of mitotic figures (absent/present) between WDSCC and PDSCC groups. A significantly higher proportion of mitotic figures was observed in PDSCC compared to WDSCC in the “present” category. Statistical analysis was performed using the Chi-square test. Data are presented as frequency and percentage. P < 0.001 indicates a statistically significant difference.

**FIGURE 3:**
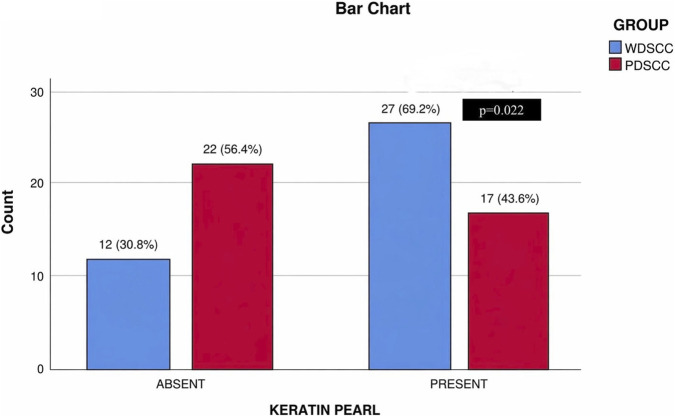
Comparison of keratin pearl formation (absent/present) between WDSCC and PDSCC groups. A significantly higher proportion of keratin pearl formation was observed in WDSCC compared to PDSCC in the “present” category. Statistical analysis was performed using the Chi-square test. Data are presented as frequency and percentage. P = 0.022 indicates a statistically significant difference.

Histopathological features revealed comparable keratinization patterns, including hyperkeratinization [21 cases (53.8%) vs. 22 cases (56.4%)] and parakeratinization [16 cases (41.0%) vs. 14 cases (35.9%)] (p = 0.921). Severe dysplasia was predominant in both groups [33 cases (84.6%) vs. 31 cases (79.5%)] (p = 0.588), and dyskeratosis was present in 26 cases (66.7%) in both groups (p = 1.000). However, mitotic figures were significantly higher in PDSCC [23 cases (59.0%)] compared to WDSCC [7 cases (17.9%)] (p < 0.001) (Figure 2). Keratin pearl formation was significantly more frequent in WDSCC [27 cases (69.2%)] than PDSCC [17 cases (43.6%)] (p = 0.022) (Figure 3).

Treatment distribution was similar in both groups, with 26 cases (66.7%) in each group receiving no treatment, while surgery was performed in 13 cases (33.3%) of WDSCC and 12 cases (30.8%) of PDSCC; only 1 case (2.6%) of PDSCC received chemoradiotherapy (p = 0.595). Follow-up data were available in 14 cases (35.9%) of WDSCC and 8 cases (20.5%) of PDSCC (p = 0.131). Overall, although most clinicopathological variables were comparable (p > 0.05), PDSCC demonstrated higher mitotic activity, increased nodal metastasis, and features associated with poorer prognosis, suggesting a trend toward more aggressive biological behavior (Table 1).

**TABLE 1:** Clinicopathological parameters.

Parameter	Category	WDSCC (n = 39)	PDSCC (n = 39)	p-value
Age	21–30	0 (0%)	1 (2.6%)	0.141
	31–40	4 (10.3%)	1 (2.6%)	
	41–50	9 (23.1%)	16 (41.0%)	
	51–60	16 (41.0%)	9 (23.1%)	
	61–70	8 (20.5%)	10 (25.6%)	
	71–80	0 (0%)	2 (5.1%)	
	81–90	1 (2.6%)	0 (0%)	
	91–100	1 (2.6%)	0 (0%)	
Sex	Male	28 (71.8%)	32 (82.1%)	0.282
	Female	11 (28.2%)	7 (17.9%)	
Medical history	Diabetes	7 (17.9%)	6 (15.4%)	0.709
	Hypertension	2 (5.1%)	4 (10.3%)	
	HIV	1 (2.6%)	1 (2.6%)	
	Anemia	1 (2.6%)	0 (0%)	
	Renal disorder	0 (0%)	1 (2.6%)	
	Combination	3 (7.7%)	1 (2.6%)	
Habits	Pan chewing	11 (28.2%)	10 (25.6%)	0.940
	Betel nut	0 (0%)	1 (2.6%)	
	Smoking	4 (10.3%)	3 (7.7%)	
	Gutkha	1 (2.6%)	1 (2.6%)	
	Combination	8 (20.5%)	9 (23.1%)	
Clinical features	Ulceroproliferative	23 (59.0%)	20 (51.3%)	0.137
	Ulcerative	10 (25.6%)	5 (12.8%)	
	Pain and swelling	3 (7.7%)	10 (25.6%)	
	Restricted mouth opening	1 (2.6%)	3 (7.7%)	
	Burning sensation	2 (5.1%)	1 (2.6%)	
Duration	Not mentioned	9 (23.1%)	15 (38.5%)	0.097
	Days	3 (7.7%)	0 (0%)	
	Weeks	1 (2.6%)	2 (5.1%)	
	Months	23 (59.0%)	22 (56.4%)	
	Years	3 (7.7%)	0 (0%)	
Laterality	Not mentioned	1 (2.6%)	1 (2.6%)	0.235
	Right	12 (30.8%)	10 (25.6%)	
	Left	26 (66.7%)	24 (61.5%)	
	Midline	0 (0%)	4 (10.3%)	
Site	Buccal mucosa	11 (28.2%)	19 (48.7%)	0.015
	Gingivobuccal sulcus	9 (23.1%)	4 (10.3%)	
	Lateral border of tongue	13 (33.3%)	3 (7.7%)	
	Retromolar trigone	1 (2.6%)	5 (12.8%)	
	Alveolar mucosa	4 (10.3%)	5 (12.8%)	
	Others	1 (2.6%)	3 (7.7%)	
Bone invasion	Absent	34 (87.2%)	35 (89.7%)	0.924
	Mandible	4 (10.3%)	3 (7.7%)	
	Maxilla	1 (2.6%)	1 (2.6%)	
Adjacent structure	Not involved	38 (97.4%)	39 (100%)	0.314
	Involved	1 (2.6%)	0 (0%)	
Skeletal muscle invasion	Absent	34 (87.2%)	31 (79.5%)	0.362
	Present	5 (12.8%)	8 (20.5%)	
Minor salivary gland	Absent	39 (100%)	36 (92.3%)	0.077
	Present	0 (0%)	3 (7.7%)	
Lymphovascular invasion	Absent	37 (94.9%)	35 (89.7%)	0.395
	Present	2 (5.1%)	4 (10.3%)	
Perineural invasion	Absent	33 (84.6%)	32 (82.1%)	0.761
	Present	6 (15.4%)	7 (17.9%)	
Metastatic lymph node	Absent	32 (82.1%)	29 (74.4%)	0.411
	Present	7 (17.9%)	10 (25.6%)	
ENE	Negative	5 (12.8%)	5 (12.8%)	0.820
	Positive	2 (5.1%)	2 (5.1%)	
Keratinization	Parakeratinized	16 (41.0%)	14 (35.9%)	0.921
	Hyperkeratinized	21 (53.8%)	22 (56.4%)	
	Others	1 (2.6%)	1 (2.6%)	
Grade of dysplasia	Moderate	5 (12.8%)	5 (12.8%)	0.588
	Severe	33 (84.6%)	31 (79.5%)	
Dyskeratosis	Absent	13 (33.3%)	13 (33.3%)	1.000
	Present	26 (66.7%)	26 (66.7%)	
Mitotic Figures	Absent	32 (82.1%)	16 (41.0%)	0.000
	Present	7 (17.9%)	23 (59.0%)	
Keratin pearl	Absent	12 (30.8%)	22 (56.4%)	0.022
	Present	27 (69.2%)	17 (43.6%)	
Treatment	Nil	26 (66.7%)	26 (66.7%)	0.595
	Surgery	13 (33.3%)	12 (30.8%)	
	Chemoradiotherapy	0 (0%)	1 (2.6%)	
Follow-up	No	25 (64.1%)	31 (79.5%)	0.131
	Yes	14 (35.9%)	8 (20.5%)	

### Immunohistochemical analysis

**FIGURE 4:**
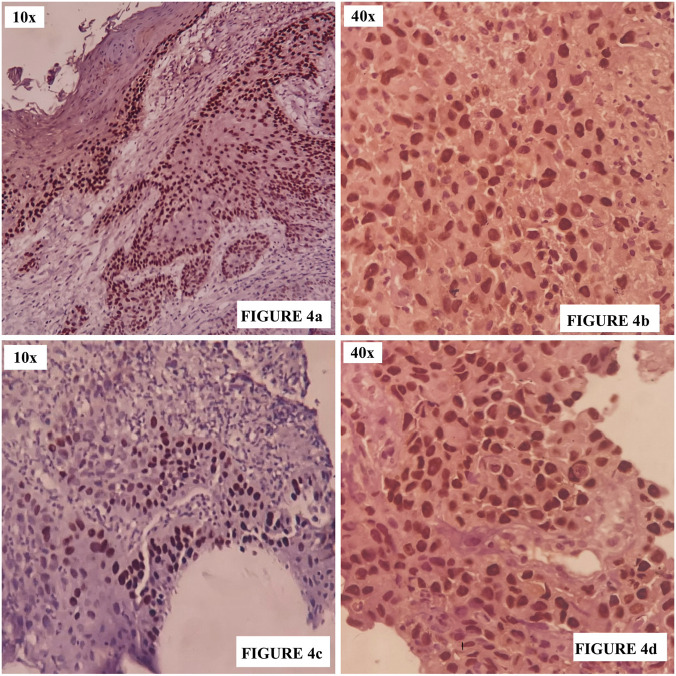
Immunohistochemical expression of p63 in oral squamous cell carcinoma **(a,b)** Well-differentiated squamous cell carcinoma (WDSCC) at 10× and 40× magnification showing strong nuclear positivity **(c,d)** Poorly differentiated squamous cell carcinoma (PDSCC) at 10× and 40× magnification showing variable nuclear positivity.

**FIGURE 5:**
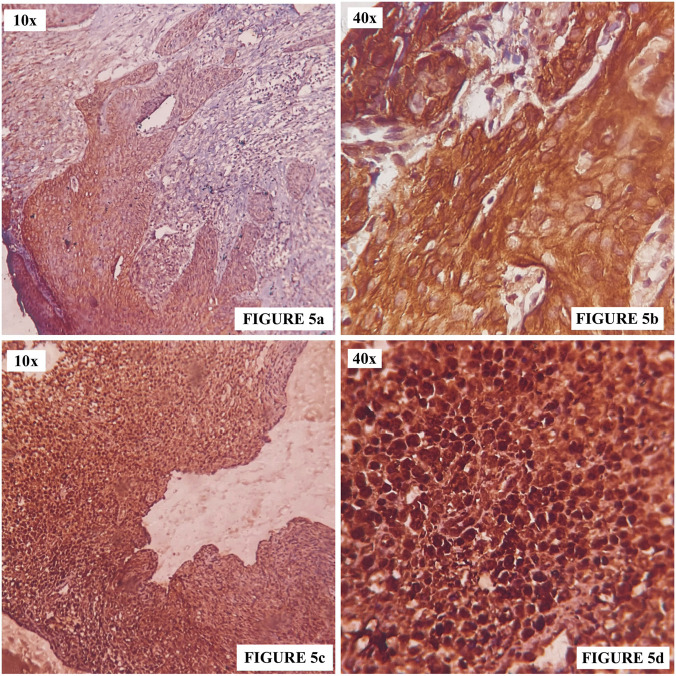
Immunohistochemical expression of EGFR in oral squamous cell carcinoma **(a,b)** Well-differentiated squamous cell carcinoma (WDSCC) at 10× and 40× magnification showing membranous staining **(c,d)** Poorly differentiated squamous cell carcinoma (PDSCC) at 10× and 40× magnification showing diffuse cytoplasmic and membranous staining.

The immunohistochemical analysis comparing EGFR and p63 expression between well-differentiated squamous cell carcinoma (WDSCC, control group) and poorly differentiated squamous cell carcinoma (PDSCC, case group), with five samples in each group, revealed distinct patterns (Figures 4, 5). For EGFR, the case group (PDSCC) suggested higher expression compared to the control group (WDSCC). The case group exhibited a median percentage of positive cells of 3 (IQR: 3–3.5), staining intensity of 3 (IQR: 2.5–3), and an immunoreactive score (IRS) of 9 (IQR: 7.5–10.5), whereas the control group showed lower values with a median percentage of positive cells of 3 (IQR: 2–3), staining intensity of 2 (IQR: 2–2.5), and an IRS of 6 (IQR: 4–7.5). However, these differences were not statistically significant (p = 0.222, 0.095, and 0.056, respectively). In contrast, p63 expression was higher in the control group (WDSCC) compared to the case group (PDSCC). The control group exhibited a median percentage of positive cells of 3 (IQR: 2.5–4) and an IRS of 9 (IQR: 6–12), whereas the case group showed lower values with a median of 1 (IQR: 0.5–2) and an IRS of 3 (IQR: 1–4.5). These differences were statistically significant (p = 0.016 for both parameters), while staining intensity did not differ significantly between groups (median three in both groups; p = 0.548) (Table 2).

**TABLE 2:** Distribution of immunohistochemical scores in WDSCC and PDSCC.

Marker	Parameter	Group 1 (Median, IQR)-WDSCC	Group 2 (Median, IQR)- PDSCC	p-value
p63	% positive cells	3 (2.5–4)	1 (0.5–2)	0.016
	Intensity	3 (2.5–3)	3 (1–3)	0.548
	IRS	9 (6–12)	3 (1–4.5)	0.016
EGFR	% positive cells	3 (2–3)	3 (3–3.5)	0.222
	Intensity	2 (2–2.5)	3 (2.5–3)	0.095
	IRS	6 (4–7.5)	9 (7.5–10.5)	0.056

### Quality of life

**FIGURE 6:**
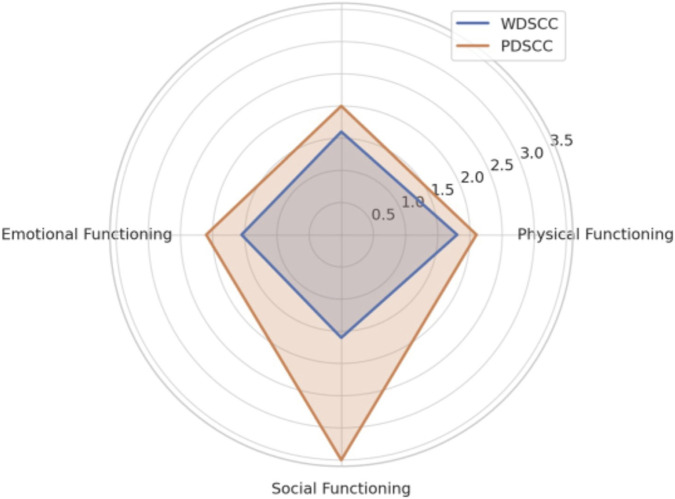
Radar plot comparing quality of life (QOL) functional domains between well-differentiated squamous cell carcinoma (WDSCC, blue) and poorly differentiated squamous cell carcinoma (PDSCC, orange). Domains assessed include physical, emotional, and social functioning.

Quality of life (QOL) assessment, performed using the EORTC QLQ-C30 and QLQ-H&N43 questionnaires, revealed substantial impairments among patients with PDSCC compared to WDSCC. Global health status scores were consistently lower in the poorly differentiated group, with significant declines in physical and role functioning domains. Symptom burden was also higher in PDSCC, with patients reporting greater severity of pain, dysphagia, xerostomia, and weight loss. Social functioning was negatively affected, with difficulties in social eating and communication being commonly reported. Psychosocial parameters reflected heightened distress, with PDSCC patients exhibiting poorer emotional wellbeing and greater financial strain due to prolonged treatment needs. Predictors of diminished QOL included advanced stage at diagnosis, invasive surgical interventions, substance use, and lack of strong social support. Collectively, these findings emphasize the dual impact of PDSCC: not only does it exhibit aggressive biological and pathological behavior, but it also significantly compromises patients’ overall quality of life (Table 3; Figure 6).

**TABLE 3:** Statistical analysis of quality of life.

Subheading	Parameter	p- value
Physical function	Trouble doing strenuous activities	0.000
	Trouble taking a long walk	0.000
	Trouble taking a short walk outside	0.000
	Need to stay in bed or chair during the day	0.002
	Need help with eating, dressing, washing, or using the toilet	0.000
	Limited in doing work or other daily activities	0.000
	Limited in pursuing hobbies or leisure activities	0.007
Symptom burden	Shortness of breath	0.001
	Experience of pain	0.000
	Trouble sleeping	0.006
	Feeling weak	0.001
	Lack of appetite	0.004
	Feeling nauseated	0.001
	Vomiting	0.001
	Constipation	0.000
	Diarrhea	0.000
	Feeling tired	0.001
	Pain interfering with daily activities	0.000
Cognitive and psychological wellbeing	Difficulty concentrating on activities like reading or watching TV	0.001
	Difficulty remembering things	0.000
	Feeling tense	0.000
	Feeling worried	0.002
	Feeling irritable	0.000
	Feeling depressed	0.000
Social and familial impact	Physical condition or treatment interfering with family life	0.000
	Physical condition or treatment interfering with social activities	0.000
	Physical condition or treatment causing financial difficulties	0.000
Oral and oropharyngeal symptoms	Problems swallowing liquids	0.000
	Problems swallowing pureed food	0.000
	Problems swallowing solid food	0.000
	Choking when swallowing	0.001
​	Problems with teeth	0.000
	Problems due to losing some teeth	0.000
	Problems opening the mouth wide	0.001
	Dry mouth	0.000
	Sticky saliva	0.000
	Problems with sense of smell	0.001
	Problems with sense of taste	0.000
	Problems with coughing	0.000
	Problems with hoarseness	0.000
Appearance and communication	Problems with appearance	0.002
	Feeling less physically attractive due to disease or treatment	0.001
	Feeling dissatisfied with body	0.001
	Problems eating	0.000
	Problems eating in front of family	0.000
	Problems eating in front of other people	0.001
	Problems enjoying meals	0.003
	Problems talking to other people	0.000
	Problems talking on the telephone	0.001
	Problems talking in noisy environments	0.000
	Problems speaking clearly	0.001
	Problems going out in public	0.001
	Feeling less interest in sex	0.001
	Feeling less sexual enjoyment	0.001
Additional physical burden	Problems raising or moving arm sideways	0.000
	Pain in shoulder	0.001
	Swelling in neck	0.000
	Skin problems (e.g., itchy, dry)	0.000
	Rash	0.001
	Skin color changes	0.000
	Worried about weight being too low	0.001
	Worried about examination results	0.000
	Worried about future health	0.000
	Problems with wound healing	0.000
	Tingling or numbness in hands or feet	0.001
	Problems chewing	0.000

### Kaplan–Meier survival analysis

**FIGURE 7:**
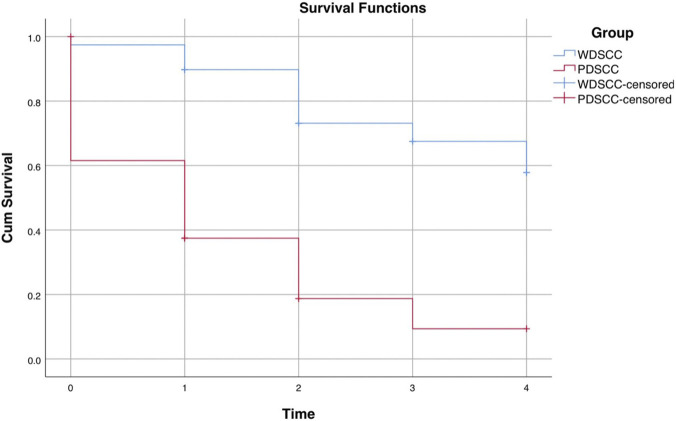
Kaplan–Meier survival analysis curves comparing well-differentiated squamous cell carcinoma (WDSCC, blue) and poorly differentiated squamous cell carcinoma (PDSCC, red). Censored cases are marked with “+”.

The Kaplan–Meier survival analysis was performed on 78 cases, including 39 WDSCC and 39 PDSCC patients. The follow-up period extended from 2019 to 2025, with a maximum follow-up duration of approximately 5 years. The follow-up duration ranged from 6 months to 5 years. Censoring was defined as patients who were alive at the end of the follow-up period or lost to follow-up. In the WDSCC group, 11 patients experienced events (death), while 28 cases were censored. In contrast, the PDSCC group showed 29 events and 10 censored cases. The mean survival time was higher in WDSCC (3.278 years; SE = 0.207; 95% CI: 2.873–3.683) compared to PDSCC (1.271 years; SE = 0.228; 95% CI: 0.823–1.718). The median survival time for WDSCC could not be estimated because more than 50% of patients were alive at the end of follow-up. In contrast, the median survival for PDSCC was 1.000 years (SE = 0.326; 95% CI: 0.361–1.639). The Log-rank (Mantel–Cox) test demonstrated a statistically significant difference between the groups (χ^2^ = 27.867, df = 1, p < 0.001), indicating significantly poorer survival outcomes in PDSCC compared to WDSCC (Table 4; Figure 7).

**TABLE 4:** Kaplan meier survival analysis.

Parameter	WDSCC	PDSCC
Mean survival (years)	3.278 (SE = 0.207)	1.271 (SE = 0.228)
95% CI (mean)	2.873–3.683	0.823–1.718
Median survival (years)	Not estimable (due to >50% censored cases)	1.000 (SE = 0.326)
95% CI (median)	Not estimable	Lower: 0.361, upper 1.639

The mean survival time was notably higher in WDSCC, compared to PDSCC., Median survival for WDSCC, could not be estimated because more than 50% of patients were alive at the end of the follow-up period. The follow-up duration ranged from 6 months to 5 years. Censored cases included patients who were alive at the last follow-up or lost to follow-up. Standard errors (SE) and confidence intervals (CI) reflect the precision of the estimates.

## Discussion

The present study highlights the aggressive clinicopathological behavior, distinct immunohistochemical profile, and compromised quality of life associated with poorly differentiated squamous cell carcinoma (PDSCC), providing insights that align with and expand upon existing literature. Clinically, our findings demonstrated that PDSCC cases presented with a shorter lesion duration and increased bone invasion compared to well-differentiated squamous cell carcinoma (WDSCC). These observations are consistent with the work of Feng et al. (2017), who emphasized that aggressive clinical features such as high lymph node ratio (LNR) and advanced clinical stage serve as significant predictors of poor prognosis in poorly differentiated carcinomas (Feng et al., 2017). Similarly, our findings of perineural and lymphovascular invasion being more frequent in PDSCC reinforce the prognostic importance of invasive features, as highlighted by Sheeba Parvez et al. (2024), (Mansoor et al., 2024). Moreover, the bone infiltration observed in our series resonates with the case report by Dharini et al. (2024), which described PDSCC with severe bone involvement and skin perforation, underscoring the destructive nature of this histological subtype (Ramalingam et al., 2024). Although PDSCC is generally considered more aggressive, bone invasion did not differ significantly between the groups in the present study, suggesting that this parameter may not independently distinguish histological grades in this cohort.

From an immunohistochemical perspective, our study suggested reduced nuclear expression of p63 in PDSCC compared to WDSCC, reflecting the loss of differentiation associated with poor prognosis. This agrees with Dayakar et al., who reported strong p63 expression in well-differentiated tumors, contrasting with its attenuation in higher-grade lesions (Dayakar et al., 2019). Likewise, EGFR expression showed an upward trend in PDSCC, although statistical significance was not reached. Importantly, Dudas et al. suggested that EGFR mislocalization from the membrane to the cytoplasm facilitates epithelial-mesenchymal transition (EMT), thereby promoting invasion and metastatic spread, a concept supported by our observation of cytoplasmic EGFR staining in PDSCC (Gao et al., 2023; Dudas et al., 2020). These findings together emphasize the diagnostic and prognostic value of IHC markers in refining the histopathological assessment of ambiguous or poorly differentiated tumors. Furthermore, recent work by Gangadhar et al. (2024) on PD-L1 expression in head and neck SCC underscores the growing relevance of immune checkpoint profiling, even though no strong correlation with clinicopathological features was demonstrated. This suggests that future studies should integrate traditional IHC markers such as p63 and EGFR with immune-based biomarkers to develop comprehensive risk stratification models (Gangadhar et al., 2024).

Quality of life (QOL) analysis in our study revealed significant impairments in patients with PDSCC, particularly in domains of physical functioning, swallowing, pain, social eating, and psychosocial wellbeing. These results echo the observations of Feng et al. (2017), who reported that the aggressive course of PDSCC, necessitating intensive interventions such as surgery and chemoradiotherapy, can substantially affect patients’ quality of life. In our study, predictors of poor QOL-including invasive surgical procedures, substance use, and limited social support, further highlight the multifactorial burden of the disease, extending beyond oncological outcomes to encompass psychosocial and financial distress. Survival analysis further emphasized the clinical impact of tumor differentiation. Kaplan-Meier curves demonstrate that WDSCC patients maintain significantly higher cumulative survival probabilities compared to PDSCC throughout follow-up, establishing a clear prognostic distinction (Kaplan and Meier, 1958). Treatment heterogeneity observed between the groups may have influenced survival outcomes and quality of life, and should be considered as a potential confounding factor while interpreting the results.

Comparisons with additional literature further strengthen the external validity of our observations. Highlighted the prognostic significance of histomorphological variants of OSCC, findings that are mirrored in our demonstration of more aggressive behavior in poorly differentiated tumors (Dhore et al., 2022). While Sharma et al. (2024) compared clinicopathological parameters between young and older OSCC patients, our data did not reveal age-specific trends, but their study parallels ours in exploring demographic and clinical modifiers of disease presentation.

One important limitation of the present study is the relatively small sample size used for immunohistochemical analysis, with only five representative cases from each group subjected to p63 and EGFR staining. While the observed expression patterns suggest potential differences in tumor differentiation and molecular behavior, the limited number of samples restricts the ability to draw definitive conclusions regarding the prognostic significance of these biomarkers. Therefore, the immunohistochemical findings should be interpreted as exploratory observations. Given the small sample size, the representativeness and generalizability of these findings are limited. Future studies incorporating larger cohorts and additional molecular markers are required to validate these preliminary findings and better define their clinical relevance in poorly differentiated squamous cell carcinoma.

Another limitation relates to the quality of life assessment conducted through telephonic interviews. Although this method allowed follow-up of patients who were otherwise difficult to access, it may introduce recall bias and subjective reporting variations. Additionally, patient responses may have been influenced by current health status, emotional condition, or the time elapsed since treatment. The variability in follow-up duration among patients may also influence the interpretation of QOL outcomes. Prospective longitudinal studies with standardized follow-up intervals and direct clinical assessments may provide more robust evaluation of quality of life outcomes in oral cancer patients. In addition, treatment modalities may act as potential confounding factors influencing survival outcomes and quality of life.

Taken together, the present study complements existing literature by being one of the few to integrate clinicopathological, immunohistochemical, and quality of life parameters in the assessment of PDSCC. By corroborating previous findings on aggressiveness, extending observations to include novel IHC interpretations, and emphasizing the importance of patient-centered outcomes, our study provides a multidimensional understanding of PDSCC that could inform both diagnostic practice and comprehensive patient care.

## Conclusion

This study highlights the aggressive nature of poorly differentiated squamous cell carcinoma (PDSCC) and shorter lesion duration compared to well-differentiated forms. Immunohistochemical analysis demonstrated reduced p63 expression, reflecting loss of differentiation, and aberrant EGFR expression, suggesting altered pathways of proliferation and invasion. Kaplan-Meier analysis showed markedly poorer overall and disease-free survival in PDSCC relative to WDSCC, highlighting its aggressive clinical course. By integrating regular follow-up and quality of life assessments, the study provides a more patient-centered perspective on disease impact. Overall, these findings reinforce the importance of early recognition, biomarker-based prognostication, and targeted management strategies, while laying a foundation for future research aimed at improving outcomes in PDSCC, although the observations are based on a relatively small immunohistochemical sample and quality of life information obtained through telephonic follow-up.

## Data Availability

The original contributions presented in the study are included in the article/supplementary material, further inquiries can be directed to the corresponding author.
